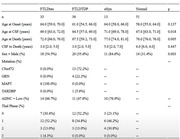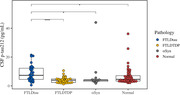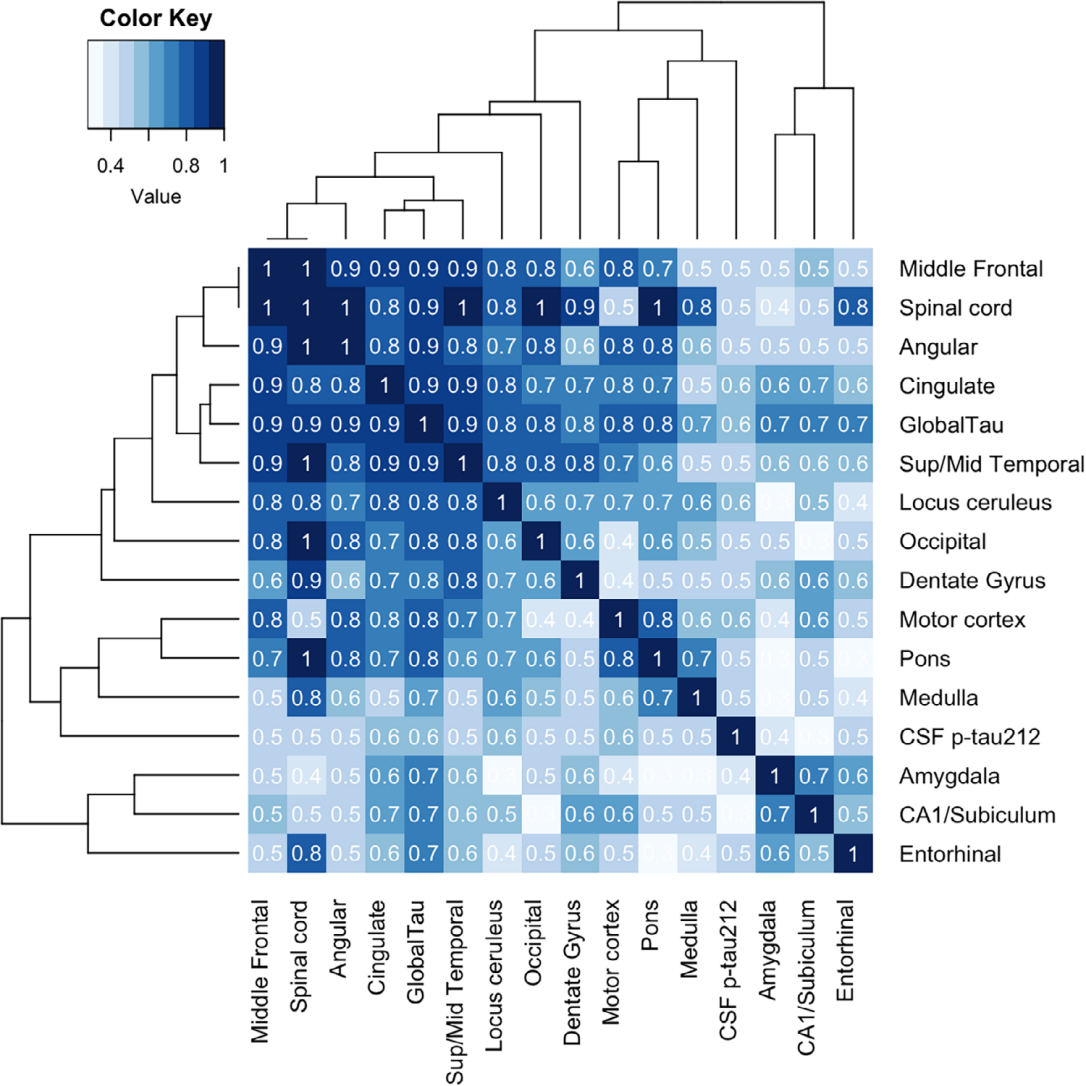# Elevated levels of CSF p‐tau212 in FTLD‐Tau

**DOI:** 10.1002/alz.090278

**Published:** 2025-01-09

**Authors:** Przemyslaw Radoslaw Kac, Katheryn A Q Cousins, Leslie M. Shaw, David J Irwin, Thomas K Karikari, Michael Turton, Vivianna M Van Deerlin, Peter Harrison, Corey T McMillan, David A Wolk, Eddie B Lee, Kaj Blennow, Henrik Zetterberg

**Affiliations:** ^1^ Department of Psychiatry and Neurochemistry, Institute of Neuroscience and Physiology, The Sahlgrenska Academy at the University of Gothenburg, Mölndal Sweden; ^2^ Department of Neurology, University of Pennsylvania, Philadelphia, PA USA; ^3^ Dept of Pathology & Laboratory Medicine, University of Pennsylvania, Perelman School of Medicine, Philadelphia, PA USA; ^4^ University of Pittsburgh School of Medicine, Pittsburgh, PA USA; ^5^ Department of Psychiatry, School of Medicine, University of Pittsburgh, Pittsburgh, PA USA; ^6^ Bioventix Plc, Farnham UK; ^7^ Center for Neurodegenerative Disease Research, Perelman School of Medicine, University of Pennsylvania, Philadelphia, PA USA; ^8^ Center for Neurodegenerative Disease Research, University of Pennsylvania, Philadelphia, PA USA; ^9^ Clinical Neurochemistry Laboratory, Sahlgrenska University Hospital, Mölndal Sweden; ^10^ Hong Kong Center for Neurodegenerative Diseases, Hong Kong China; ^11^ Department of Neurodegenerative Disease and UK Dementia Research Institute, UCL Institute of Neurology, Queen Square, London UK; ^12^ Wisconsin Alzheimer’s Disease Research Center, University of Wisconsin School of Medicine and Public Health, Madison, WI USA; ^13^ Dementia Research Centre, Department of Neurodegenerative Disease, UCL Queen Square Institute of Neurology, University College London, London, United Kingdom, London UK

## Abstract

**Background:**

Phosphorylated‐tau (p‐tau) biomarkers are typically specific for Alzheimer’s disease (AD) and are less elevated in the cerebrospinal fluid (CSF) of frontotemporal lobar degeneration (FTLD) type tau (FTLD‐tau). FTLD is a pathologically and clinically heterogenous neurodegenerative disorder, and we currently lack biomarkers to differentiate the two major pathological subtypes 1) FTLD‐tau, where pathological tau aggregation is observed or 2) FTLD‐TDP, where TAR‐DNA binding protein 43 (TDP‐43) is linked with the disease development and progression. Because FTD clinical phenotype does not predict pathology, biomarkers sensitive to FTLD‐tau are needed to provide a biological diagnosis in life. In this study, we test CSF p‐tau212 in patients with autopsy‐ or mutation‐confirmed FTLD‐tau and FTLD‐TDP without AD pathology; clinically healthy individuals without cognitive impairment and autopsy‐confirmed Lewy body disease with alpha‐synuclein (αSyn) were included as reference groups.

**Method:**

Autopsies were performed at the University of Pennsylvania in the Center for Neurodegenerative Disease Research. Definite diagnosis was based on autopsy results or determined genetically. Using SIMOA technology, we tested p‐tau212 in 51 healthy controls, 33 FTLD‐Tau, 36 FTLD‐TDP and 13 αSyn (Figure 1). For further analysis we excluded individuals with evidence of AD pathology: intermediate/high AD neuropathologic change (ADNC), Thal phase ≥2, or CSF β‐amyloid 1‐42 ≤192. Wilcoxon test was used to compare groups. In brain regions sampled at autopsy, pathological tau burden was rated using a semi‐quantitative 5‐point scale; Spearman correlations tested associations between p‐tau212 and tau burden by region.

**Result:**

CSF p‐tau212 was significantly increased in FTLD‐Tau when compared to healthy controls, people with FTLD‐TDP and people with αSyn pathology (Figure 2). Results remained significant when people with evidence of AD pathology were excluded. P‐tau212 measurements strongly correlated with tau burden across multiple brain regions (Figure 3). Average correlation was r=0.57, p<0.0001.

**Conclusion:**

CSF p‐tau212 is strong candidate biomarker to differentiate FTLD‐tau from FTLD‐TDP and αSyn.